# *Lysiphyllum strychnifolium* (Craib) A. Schmitz Extracts Moderate the Expression of Drug-Metabolizing Enzymes: In Vivo Study to Clinical Propose

**DOI:** 10.3390/ph16020237

**Published:** 2023-02-03

**Authors:** Natthaporn Kuendee, Alisa Naladta, Thitianan Kulsirirat, Thunyatorn Yimsoo, Werayut Yingmema, Kanoktip Pansuksan, Korbtham Sathirakul, Sophida Sukprasert

**Affiliations:** 1 Faculty of Veterinary Medicine, Rajamangala University of Technology Tawan-ok, Chonburi 20110, Thailand; 2 Department of Biochemistry, Faculty of Science, Khon Kaen University, Khon Kaen 40002, Thailand; 3 Department of Pharmacy, Faculty of Pharmacy, Mahidol University, Bangkok 10400, Thailand; 4 Animal Center, Thammasat University (Rangsit Campus), Pathum Thani 12120, Thailand; 5 Chulabhorn International College of Medicine, Thammasat University (Rangsit Campus), Pathum Thani 12120, Thailand

**Keywords:** alcohol detoxification, cytochrome P450, CYP induction, CYP inhibition, *Lysiphyllum strychnifolium*

## Abstract

*Lysiphyllum strychnifolium* (Craib) A. Schmitz (LS) has been traditionally used as a medicinal herb by folk healers in Thailand with rare evidence-based support. Hepatic cytochrome P450s (CYPs450) are well known as the drug-metabolizing enzymes that catalyze all drugs and toxicants. In this study, we investigated the mRNA levels of six clinically important CYPs450, i.e., CYP1A2, 3A2, 2C11, 2D1, 2D2, and 2E1, in rats given LS extracts. Seventy Wistar rats were randomized into seven groups (n = 10). Each group was given LS stem ethanol (SE) and leaf water (LW) extracts orally at doses of 300, 2000, and 5000 mg/kg body weight (mg/kg.bw) for twenty-eight consecutive days. After treatment, the expression of CYPs450 genes was measured using quantitative real-time PCR. The results revealed that SE and LW, which contained quercetin and gallic acid, promoted the upregulation of all CYPs450. Almost all CYPs450 genes were downregulated in all male LW-treated rats but upregulated in female-treated groups, suggesting that CYP gene expressions in LS-treated rats were influenced by gender. Moderate and high doses of the LS extracts had a tendency to induce six CYP450s’ transcription levels in both rat genders. CYP2E1 gene showed a unique expression level in male rats receiving SE at a dose of 2000 mg/kg.bw, whereas a low dose of 300 mg/kg.bw was found in the LW-treated female group. As a result, our findings suggest that different doses of LS extracts can moderate the varying mRNA expression of clinically relevant CYP genes. In this study, we provide information about CYP induction and inhibition in vivo, which could be a desirable condition for furthering the practical use of LS extracts in humans.

## 1. Introduction

The use of herbal medicines as complementary or alternative medicines has become increasingly common in many countries worldwide due to scientific support for their therapeutic effectiveness and fewer harmful side effects than conventional therapies [1,2]. *Lysiphyllum strychnifolium* (Craib) A. Schmitz (Fabaceae family) (LS) is also known as Ya nang daeng or Khayan [3]. It is a common medicinal plant found in many regions of Thailand. This plant is a popular folk medicine that has traditionally been used for a variety of medical purposes such as treating fever, cancer, allergy, and alcohol intoxication, as well as for detoxification, promoting health, and neutralizing pesticide poisoning in animals and humans [3,4,5]. The stem ethanol extract of LS has been used for anti-HIV-1 integrase and showed anti-allergy properties [6] and antidiabetic properties [7]. The dried powdered stems and leaves are applied as herbal tea, and hydroalcoholic extracts are widely prescribed by traditional doctors [4]. Active ingredients derived from the LS extracts are mainly composed of polyphenols and flavonoids with broad biological and pharmacological activities [3,5,8]. Bunluepuech et al. reported on the isolation of five compounds from the stem ethanol extract of LS, i.e., quercetin (Que), 3,5,7,3’,5’- pentahydroxyflavanonol-3-O-α-L-rhamnopyranoside,3,5,7-trihydroxychromone-3-α-L-rhamnopyranoside, sitosterol, and stigmasterol [6], and astilbin was also isolated from the stem methanol extract [9]. Corresponding to our previous studies, we reported that Que derived from the LS stem ethanol extract had anti-H5N1 and antidote activities in omethoate-treated rats [5,6]. In the LS leaf water extract, three bioactive components, Yanangdaengin, trilobatin [10], and gallic acid (GA), were identified [3,10].

CYP450 is primarily expressed in the liver and is well known as a key enzyme in phase-I metabolism, detoxifying all toxicants [11]. Therefore, the co-consumption of the herb and drug can mediate CYP induction and inhibition, resulting in unexpected effects (i.e., efficacy loss or increased toxicity) due to the interference of pharmacological effects between herbal products and drugs, which is defined as “herb–drug interactions” (HDIs) [12,13,14]. HDIs mostly depend on the effects of CYP450 and drug transporters, i.e., p-glycoprotein (p-gp). The expression of CYPs450 and p-gp is regulated by various transcription factors, such as nuclear receptors such as the pregnane X receptor (PXR) and constitutive androstane receptor (CAR), or basic helix-loop-helix/Per-Arnt-Sim such as AhR, which are activated by specific endogenous or exogenous ligands [15]. HDIs occur in the liver and in the gastrointestinal epithelium due to the presence of CYP450 enzymes [16]. They can occur on both a pharmacodynamic and pharmacokinetic basis [17].

Nowadays, the World Health Organization (WHO) is concerned about HDIs because the frequency of using herbal medicine (HM) has increased in countries worldwide. The WHO has reported that approximately 80% of the world’s population is using herbal products for health benefits and disease treatment purposes, particularly in developing countries where HM is frequently utilized as a primary source of healthcare treatment [18,19]. Regarding the rising demand and practice of medicinal plants in the general public, wide-ranging research concerning their efficacy and safety when used with conventional medicines is important [17,20]. Herbal plants utilize the same metabolic pathways and transporter systems as drugs for absorption, distribution, metabolism, and excretion processes in the body. Normally, HMs are generally assumed to be modulators, inhibitors, or inducers when evaluating HDIs. There are two main ways in which HMs contribute to HDIs. Firstly, the influence of herbal products on drug transporters or metabolic enzymes: the inhibition of transporters leads to decreased transporter-mediated efflux of drugs and effectively increases the absorption of a drug [14]. Secondly, herbal products alter the synergistic effect of metabolic transporters and metabolic enzymes [21]. Some herbal products, such as garlic, ginkgo, ginseng, and grape juice, act as both P-gp and CYP3A inhibitors [13,22,23,24], resulting in increased drug concentrations in the liver and plasma [14,24]. The combination of medicinal plants with conventional drugs is used by up to 35% of patients [25]. For example, Clairet et al. reported that 30–70% of cancer patients use alternative medicines during chemotherapy [26]. The prevalence of HM use in people living with HIV ranges from 50–95%, whereas HM is also of great significance for the treatment of tuberculosis [27,28]. Previous studies revealed that HDIs differ from HM in terms of their interaction potential. *Ginkgo biloba* amentoflavone metabolite is a potent inhibitor of CYP2C9 and CYP3A4 activities [29], Que in *Allium cepa* has been shown to inhibit P-gp and CYP3A4 [24], and Que in *Allium sativum* inhibits CYP2E1 [30]. *Momordica charantia* contains P-glycopeptide, sterol glucosides, charantin, and vicine, all of which inhibit CYP2C9 [31].

Currently, folk healers, farmers, and the rural population in Thailand are now consuming LS and its extracts as HM. Along with conventional medicine, it has been used to treat diseases, maintain health, and promote well-being. Despite the safety of such supplements, their use is increasing due to a lack of knowledge about the safety and potential effect of extracts on CYPs450 in the liver, the most important metabolizing enzyme that may influence HDIs [32,33]. Herein, we aimed to investigate the action of leaf water (LW) and stem ethanol (SE) extracts of LS on the drug-metabolizing enzyme mRNA expression of six CYPs450 gene isoforms, i.e., CYP1A2, CYP3A2, CYP2C11, CYP2D1, CYP2D2, and CYP2E1, in rats to elucidate the effects of this herb on clinical implications in the future. Furthermore, the cytotoxicity of both LS extracts, pure Que and GA, on HepG2 cells was determined. 

## 2. Results

### 2.1. Determination of Que and GA in LS Extracts

**Figure 1:**
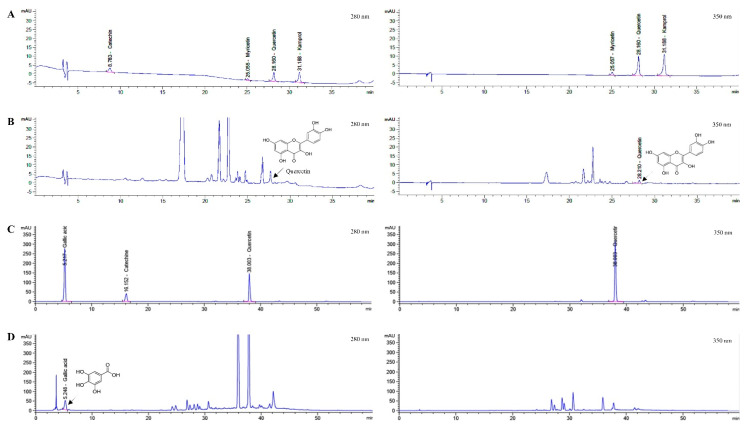
Representative HPLC chromatogram of the standards (**A,C**), SE (**B**), and LW (**D**). The Que and GA peaks are indicated by the arrows.

Chromatographic profiles of the two LS extracts, SE and LW, were detected using HPLC-DAD. Five standards, i.e., Que, kaempferol, myricetin, catechin, and GA, were used (Figure 1A,C). The Que and GA derived from the SE and LW were detected at 280 and 350 nm, as indicated by the arrows (Figure 1B,D).

### 2.2. Cytotoxicity Test

**Figure 2:**
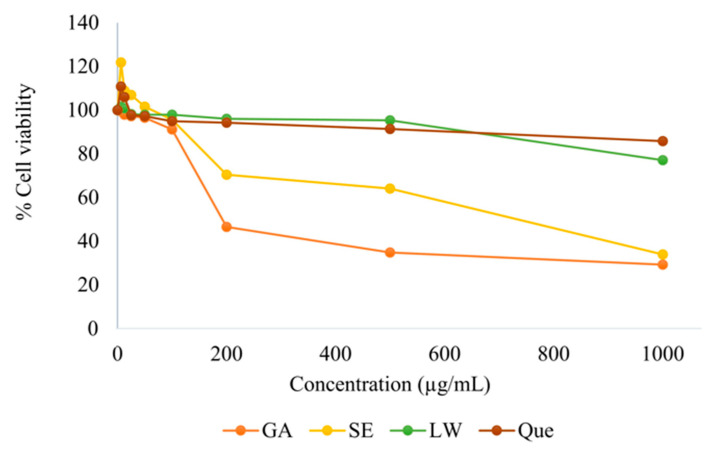
Cytotoxicity of the LS extracts, standard Que, and GA was investigated in HepG2 cells after incubation at 37 °C for 24 h determined by the 3-(4,5-dimethylthiazol-2-yl)-2,5-diphenyltetrazolium bromide (MTT) assay. The data represent the mean ± standard deviation (SD) of triplicates.

As shown in Figure 2, the LS extracts GA and Que were nontoxic to HepG2 cells at concentrations below 100 µg/mL with a percentage cell viability greater than 90%. GA and SE at a concentration of 500 µg/mL showed cytotoxicity of 35% and 64%, respectively, whereas the non-cytotoxicity of LW and Que was still greater than 90% at the same concentration. However, the cell viabilities of the SE, LW, and Que at 1000 µg/mL were 34%, 77%, and 86%, respectively. This result indicated that the LS extracts that showed cell viability greater than 50% were safe for further study in animals.

### 2.3. Effect of SE on Hepatic mRNA CYPs450

**Figure 3:**
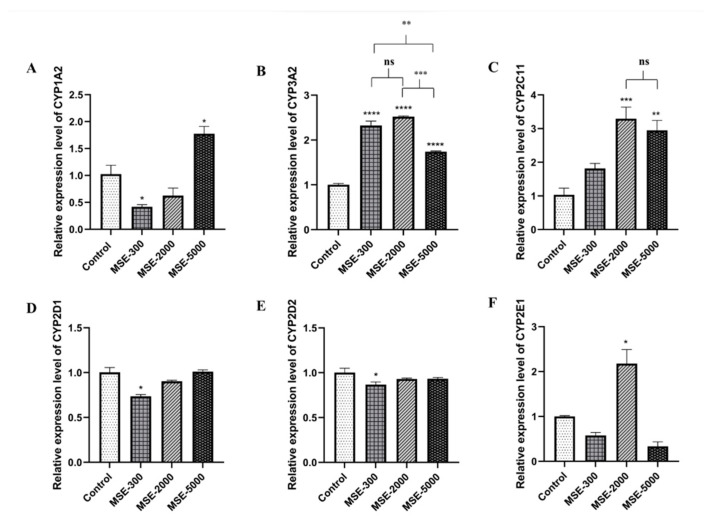
Relative expression levels of CYPs450 mRNA in male Wistar rats orally treated with SE at 300, 2000, and 5000 mg/kg.bw/day or the control (distilled water) for 28 days. Relative gene expressions of CYP1A2 (**A**), CYP3A2 (**B**), CYP2C11(**C**), CYP2D1 (**D**), CYP2D2 (**E**), and CYP2E1 (**F**) were normalized to that of GAPDH. The results are presented as the mean ± the Standard Error of the Mean (SEM). * *p* < 0.05, ** *p* < 0.01, *** *p* < 0.001, and **** *p* < 0.0001 versus the control group; ns, not significant. MSE-300, -2000, and -5000 represent male rats treated with SE at doses of 300, 2000, and 5000 mg/kg.bw/day, respectively.

**Figure 4:**
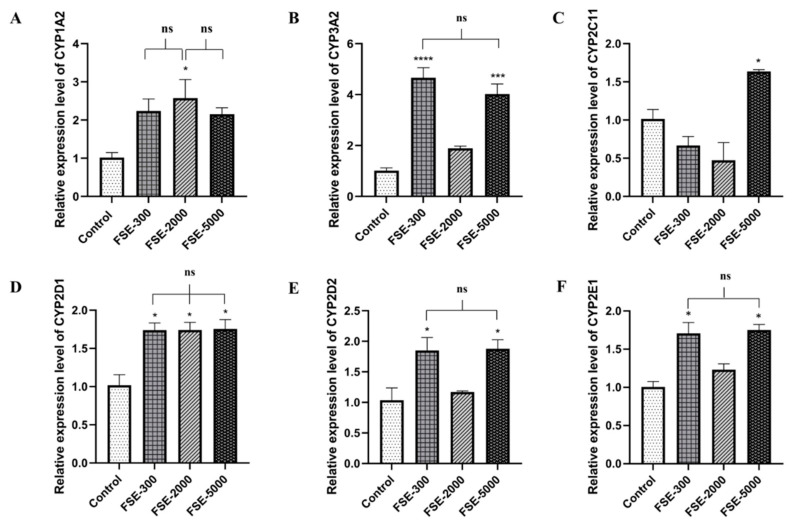
Relative expression levels of CYPs450 mRNA in female Wistar rats orally treated with SE at 300, 2000, and 5000 mg/kg.bw/day or the control (distilled water) for 28 days. Relative gene expressions of CYP1A2 (**A**), CYP3A2 (**B**), CYP2C11(**C**), CYP2D1 (**D**), CYP2D2 (**E**), and CYP2E1 (**F**) were normalized to that of GAPDH. The results are presented as the mean ± SEM. * *p* < 0.05, *** *p* < 0.001, **** *p* < 0.0001 versus the control group; ns, not significant. FSE-300, -2000, and -5000 represent female rats treated with SE at doses of 300, 2000, and 5000 mg/kg.bw/day, respectively.

Figure 3 and Figure 4 show the effects of three SE doses on the mRNA expression of six CYPs450: 1A2, 3A2, 2C11, 2D1, 2D2, and 2E1 in male (MSE) and female (FSE) SE-treated rats. At the low dose of 300 mg/kg body weight (mg/kg.bw), among the six CYP isoforms, CYP1A2 was significantly decreased by 0.41-fold, whereas at the dose of 5000 mg/kg.bw of the extract, it was markedly increased by 1.73-fold compared to the control group (Figure 3A). CYP3A2 was significantly upregulated in all SE-treated groups. The increase was 2.32-, 2.52-, and 1.74-fold, respectively, relative to that in the control (*p* < 0.0001) (Figure 3B). Upregulation of CYP2C11 was observed in the group treated with 2000 and 5000 mg/kg.bw, but not in the 300 mg/kg.bw-treated group (Figure 3C). Furthermore, the CYP2D1 and D2 isoforms showed no significant difference in the SE-treated groups at medium and high doses but were downregulated in the low dose-treated group compared to the control (Figure 3D,E). Interestingly, among the six CYP isoforms, CYP2E1 in the medium MSE-treated group (2000 mg/kg.bw) was highly upregulated by 2.18-fold compared to the control (Figure 3F). 

For FSE, CYP1A2 seemed to increase at all doses in the SE-treated group. However, only a dose of 2000 mg/kg.bw resulted in a significant difference (*p* < 0.05) of 2.35-fold compared to the control group (Figure 4A). The other CYP isoforms (CYP3A2, 2D2, and 2E1) demonstrated significantly increased mRNA levels at 300 and 5000 mg/kg.bw compared to the control groups as shown in Figure 4B,E,F. CYP2C11 showed dominantly high expression in the high-dose SE-treated group compared to the other groups (Figure 4C). Additionally, the expression of CYP2D1 was highly expressed at all doses, with no statistically significant difference among all SE-treated groups. However, all SE-treated groups were observed to increase when compared to the control group (*p* < 0.05) (Figure 4D).

These findings indicated that different SE doses influenced the up- and down-regulation of the six clinically relevant CYPs450s. Furthermore, the SE had an effect on the expression level of sex-based CYPs450.

### 2.4. Effect of LW on Hepatic mRNA CYPs450

**Figure 5:**
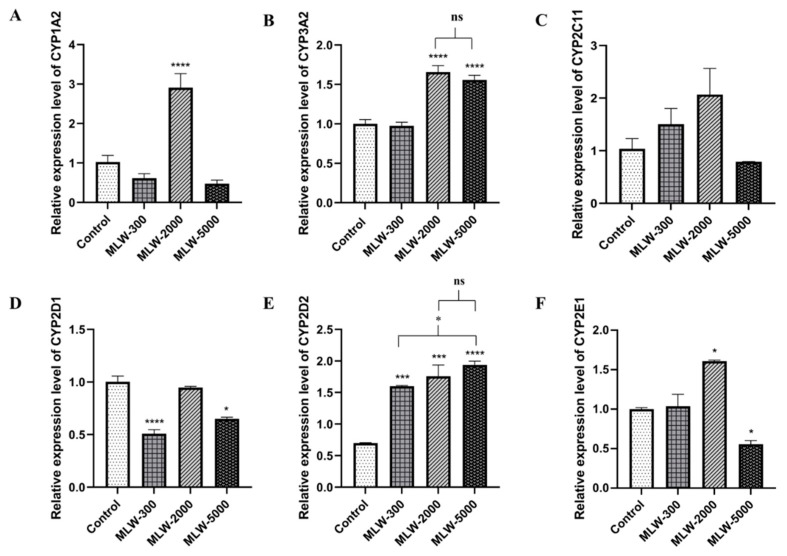
Relative expression levels of CYPs450 mRNA in male Wistar rats orally treated with LW at 300, 2000, and 5000 mg/kg.bw/day or the control (distilled water) for 28 days. Relative gene expressions of CYP1A2 (**A**), CYP3A2 (**B**), CYP2C11(**C**), CYP2D1 (**D**), CYP2D2 (**E**), and CYP2E1 (**F**) were normalized to that of GAPDH. The results are presented as the mean ± SEM. * *p* < 0.05, *** *p* < 0.001, and **** *p* < 0.0001 versus the control group; ns, not significant. MLW-300, -2000, and -5000 are male rats treated with LW at doses of 300, 2000, and 5000 mg/kg.bw/day, respectively.

**Figure 6:**
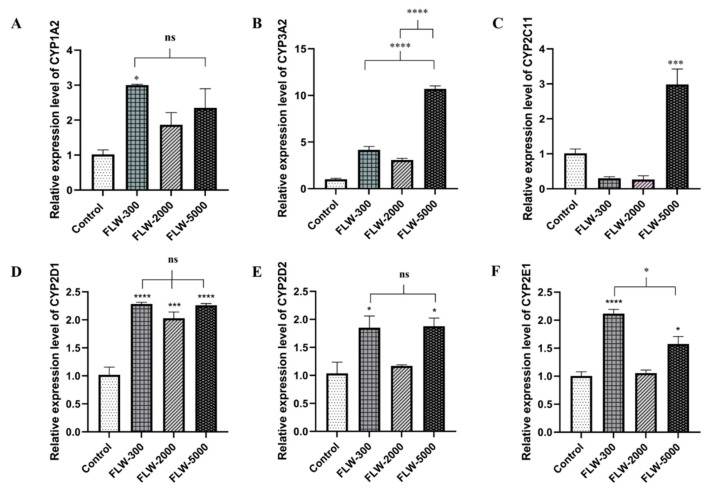
Relative expression levels of CYP450s mRNA in female Wistar rats orally treated with LW at 300, 2000, and 5000 mg/kg.bw/day or the control (distilled water) for 28 days. Relative gene expressions of CYP1A2 (**A**), CYP3A2 (**B**), CYP2C11(**C**), CYP2D1 (**D**), CYP2D2 (**E**), and CYPE1 (**F**) were normalized to that of GAPDH. The results are presented as the mean ± SEM. * *p* < 0.05, *** *p* < 0.001, and **** *p* < 0.0001 versus the control group; ns, not significant. FLW-300, -2000, and -5000 are female rats treated with LW at doses of 300, 2000, and 5000 mg/kg.bw/day, respectively.

Figure 5 and Figure 6 present the expression levels of six clinically relevant CYPs450 in male (MLW) and female (FLW) LW-treated rats. The results showed that CYP1A2 was highly expressed by 2.84-fold (*p* < 0.0001) in the MLW-treated group at the dose of 2,000 mg/kg.bw compared to the control group (Figure 5A). Figure 5B shows that CYP3A2 mRNA expression was significantly higher in the MLW-2000- and MLW-5000-treated groups than in the control group (*p* < 0.0001). There were no significant differences in CYP2C11 mRNA expression between all MLW-treated groups and the control (Figure 5C). The levels of mRNA expression for CYP2D1 in MLW-300 and MLW-5000 were distinctly lower than those of the control group (*p* < 0.0001, 0.05) by 0.49- and 0.35-fold, respectively (Figure 5D). In contrast, the CYP2D2 mRNA levels were upregulated and significantly higher than those in the control group (Figure 5E). In addition, CYP2E1 was upregulated in the MLW-2000-treated group but downregulated in the MLW-5000-treated group (*p* < 0.05) (Figure 5F). 

In the FLW groups, the results revealed that CYP1A2 had a tendency to be highly expressed in the high-dose LW-treated group (Figure 6A). The CYP3A2 mRNA levels were not significantly different at all doses of the LW-treated groups compared to the control (Figure 6B). CYP2C11 at the high dose in the LW-treated group was markedly increased by 2.94-fold compared to the control (Figure 6C). Interestingly, the CYP2D1 levels in all treated groups were significantly increased by 2.25-, 2.00-, and 2.22-fold relative to those in the control group (Figure 6D). Meanwhile, the mRNA expression of CYP2D2 and 2E1 mRNA at low and high doses were distinctly increased compared with controls; in particular, the CYP2E1 level in the 300 mg/kg.bw-treated group had a twofold higher transcription level than the control group (Figure 6E,F).

### 2.5. Heat Map Analysis 

**Figure 7:**
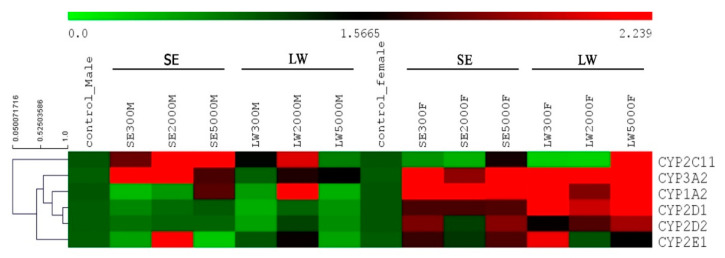
Heat map of the relative expressions of CYP1A2, CYP3A2, CYP2C11, CYP2D1, CYP2D2, and CYP2E1 in male and female SE/LW-treated rats. The bright red color indicates a high value of gene expression in each treatment, whereas the lowest gene expression is presented in bright green, as shown in the color scale bar above the heat map.

In this study, heat map analysis was also used to emphasize and clearly show the mRNA expression levels of the six CYP isoforms. The default color gradient sets the lowest value in the heat map to bright green, the highest value to bright red, and mid-range values to black, with a corresponding transition (or gradient) between these extremes; the data are shown in Figure 7. In the MSE-treated groups, the results revealed that CYP2C11 was most dominantly expressed at the doses of 2000 and 5000 mg/kg.bw, whereas CYP3A2 showed a high expression at doses of 300 and 2000 mg/kg.bw. Interestingly, MSE at a dose of 2000 mg/kg.bw upregulated CYP2E1 compared to the others. In the FSE-treated groups, CYP3A2 and CYP1A2 were dominant and highly upregulated at all doses. By contrast, in the MLW-treated groups, only CYP2C11 and CYP1A2 were more highly expressed than the others at a dose of 2000 mg/kg.bw. In the FLW-treated groups, three CYP isoforms, i.e., CYP3A2, CYP1A2, and CYP2D1, were upregulated at all treated doses, except CYP2C11 at the high dose. The expression of CYP2D2 had a tendency to increase at all doses. Furthermore, CYP2E1 was upregulated at low doses of LW, but it was inhibited at high doses of LW. These findings suggested that different doses of the two LS extracts affected different CYPs450, implying their potential role in drug administration in future clinical trials.

## 3. Discussion

In the last decade, the number of people who use herbal treatments as complementary and/or alternative medicine to conventional medicine has increased. Although it has been established that several active compounds in herbal plants have the potential to modulate the activity of drug-metabolizing enzymes, to ensure the safety of herbal products, we demonstrated for the first time the effect of the LS-SE and LS-LW on the hepatic mRNA expressions of the six clinically relevant CYPs450 isoforms (CYP1A2, 3A2, 2C11, 2D1, 2D2, and 2E1) in animals. The principal findings of this study include the following: in particular, (i) six CYPs450 enzymes were induced and inhibited by LS extracts used as herbal supplements; (ii) the mRNA expressions of most CYPs450 were influenced by gender. Almost all CYPs450 genes were downregulated at all MLW doses but upregulated in the female-treated groups; (iii) in female-treated rats, SE and LW induced CYP 3A2 and 1A2 in all doses; and (iv) moderate and high doses of the LS extracts tended to induce six CYP450s’ transcription levels in female-treated rats.

The most regular mechanisms of herb extracts imply the induction or inhibition of CYP450 expression, which correlates with protein synthesis [34] and enzyme activity [2]. The stimulation of CYP450 enzymes enhances drug clearance, leading to a decrease in the therapeutic effect, while inhibition results in a decrease in drug excretion and increases the drug plasma concentration, causing toxicity and therapeutic failure [2,15]. In this study, we reported the Que and GA derived from LS-SE and LS-LW. Que is a member of the flavonoid family and is one of the most prominent dietary antioxidants. It is ubiquitously present in plants and human diets, as well as in countless foods. Que and its derivatives derived from the LS have also been reported in previous studies [16,17,18,19,20]. These naturally occurring flavonoids have been shown to modulate the CYP450 enzymes’ activity by various mechanisms, including through specific receptor genes and/or CYP protein alteration or mRNA stabilization [21]. However, GA derived from the LW of the LS was also identified. Previous studies have reported that GA, Yanangdaengin, and trilobatin are compounds found in the LS water extract [3,10,35].

CYP1A2 accounts for 13% of the total CYP content and is involved in the metabolism of 8–10% of the drugs on the market [36,37,38]. This enzyme is well confirmed as a potential cancer-promoting agent in colorectal, breast, and lung cancers [39]. Our study showed that hepatic CYP1A2 mRNA expression at MSE-300 mg had a predominant inhibitory effect, while other concentrations in both genders revealed a tendency to up-regulate. Moreover, Xiao et al. reported that CYP1A2 enzyme activity can be induced by smoking [40]. As a result, the practical application of LS extracts should be concerned with dose adjustment, particularly in the male smoking population. In addition, the combined treatment of LS-SE and substrate drugs metabolized by human CYP1A2 may result in significant HDIs.

CYP3A is the most abundant CYP enzyme in the human liver. It is involved in the biotransformation of numerous endogenous substances, such as fatty acids, eicosanoid sterols, vitamin D4, and bile ducts. It participates in the metabolism of more than 75% of clinical drugs [41] and plays a considerable role in the metabolism of a range of pharmaceutical products [42]. Our study revealed that CYP3A2 induction was stimulated at all doses of both extracts in female-treated rats. CYP inhibition was also investigated in male-LW treated rats. The results suggest that CYP3A2 is affected by the components present along with LS extracts, which may modulate the transcription of CYP3A2. The evidence of a stimulation effect on CYP3A2 from a herbal extract in which Que was one of the major components is consistent with previous observations revealing that the mRNA expression levels of CYP3As were induced by Que [43]. This induction effect of CYP3A2 (CYP3A4 in humans), a detoxifying enzyme, has been found for anti-cancer chemotherapy drugs such as docetaxel, vincristine, and paclitaxel [44]. All drugs are susceptible to clinically relevant pharmacokinetic interactions with their respective metabolizing enzymes. Therefore, anticancer agent co-administration with LS extract should raise awareness about dose adjustment because increasing CYP2A3 levels have the potential to increase the drug’s plasma concentration, potentially leading to toxicity.

Rat CYP2C11 is functionally similar to human CYP2C9 [2]. CYP2C11 has a proportion of 60% of the total; it metabolizes approximately 15% of all drugs [45] and is considered the main enzyme for the anti-coagulant warfarin [46], the anti-diabetic agent tolbutamide, the nonsteroidal anti-inflammatory drug diclofenac [45], angiotensin II blockers, and antiepileptics [47]. Our findings showed that male rats given SE and LW at moderate and high doses promoted CYP2C11 induction; the same response was observed only at the highest dose in female LW-treated rats. Therefore, the co-administration of LS extracts at moderate and high doses with drugs metabolized by CYP2C11 may increase elimination and reduce the therapeutic effect. For example, the co-consumption of tolbutamide, a type 2 diabetes medicine, with LS extract higher than 2000 mg/kg.bw/day may diminish the drug’s concentration in plasma and reduce its effect. Thus, moderate and high concentrations of LS extracts should not be consumed by diabetic patients, given their stimulatory action on the CYP2C9 metabolic enzyme [48], and practical use, which may require dose adjustment to avoid undesirable HDIs.

Rat CYP2D1 and 2D2 are the orthologs of human CYP2D6 and are generally known as CYP2D. Approximately 20–50% of clinical drugs are metabolized by CYP2D [49]. In vivo clearance of CYP2D6 substrates in poor metabolizers is generally much lower than that in extensive metabolizers, leading to higher plasma concentrations and the potential for clinical toxicities at therapeutic doses [45]. Our results showed that males treated with the medium and high doses of SE and LW had no effects on CYP2D1 and 2D2, whereas the mRNA expression levels of female rats tended to increase. Our finding suggests that the use of products containing LS may be considered safe when co-administered with CYP2D substrates such as basic lipophilic nitrogen-containing molecules and alkaloids [50] and has the ability to reduce clinical toxicities from therapeutic drug doses.

Rat CYP2E1 is a homolog of human CYP2E1, and it displays a substrate preference for various molecules, including ethanol [51]. Hence, CYP2E1 is one of the candidate genes for alcohol dependence [52] and plays an important role in rapidly reversing alcohol withdrawal [53] and elevating alcohol concentrations after chronic consumption due to induction [51,54]. We found that the lowest dose of LW promoted CYP2E1 induction in female-treated rats, whereas the medium dose of SE resulted in similar findings in male-treated rats. As a result, LS extracts, particularly LS-LW, which is commonly inhaled as herbal tea, are very interesting to be used as herbal medicine for rapidly clearing alcohol consumption in the female drinker population. Moreover, a previous study demonstrated that the inhibitory potential of CYP2E1 has the ability to boost the clearance of anti-cancer agents such as docetaxel and of the antiretroviral drug saquinavir [30]. As a result, in the future, LS extracts may be used as HM or as a drug supplement for cancer patients who smoke. 

## 4. Materials and Methods

### 4.1. Plant Materials and Extraction

The LS stems and leaves were collected in 2019 from Naso subdistrict, Kut Chum district, Yasothon Province, Thailand, between June and July, with a harvest time of 120–180 days. It was taxonomically identified (a voucher specimen TTM no.0003601) for confirmation at the Department of Thai Traditional and Alternative Medicine’s Herbarium in Thailand. Dried powders of LS stems and leaves were extracted using ethanol and water, respectively, following the protocol from our previous study [8]. Ethanol from the SE was removed by vacuum evaporation, while the LW was spray dried. Both dried extracts were then stored in a sealed container at -20 °C for further analysis.

### 4.2. Chemicals and Reagents

Que (purified ≥ 98%), kaempferol, myricetin, catechin, and GA were purchased from Sigma (St. Louis, MO, USA). Water used in this study was purified using the Milli-Q water purification system (WATER PRO^®^ PS, Missouri, USA). All other chemicals were analytical grade and were purchased from local chemical suppliers. HPLC-grade solvents obtained from Fisher Scientific (Seoul, Korea) were used. Dulbecco’s modified Eagle’s medium (DMEM) was purchased from Gibco^®^ (Invitrogen, USA). HBSS trypsin–EDTA solution, and penicillin and streptomycin solution were purchased from GibcoTM (Life Technologies, Grand Island, NY, USA). Sodium pyruvate solution was purchased from Calbiochem^®^ (Merck, Germany). Fetal bovine serum (FBS) was purchased from Hyclone^®^ (GE Healthcare Life Sciences, Utah, USA). 

Trizol reagent (Invitrogen, Camarillo, CA, USA) and the ReverseAid First-Strand cDNA Synthesis Kit (BioRad, Hercules, CA, USA) were used for RNA extraction and cDNA synthesis. Forward and reverse primers for the CYP450 genes were synthesized for mRNA expression level determination (Pacific Science Company, Bangkok, Thailand). Maxima SYBR Green qPCR master mix (Westburg Life Sciences, The Netherlands) was used for real-time PCR (RT-PCR) reactions.

### 4.3. HPLC Analysis 

HPLC was performed following a modified protocol from our previous study [5]. Briefly, the HPLC conditions for SE were as follows: Thermo Hypersil gold C18 RP column (Thermo Fisher Scientific, San Jose, CA, USA: 250 mm × 2.1 mm i.d., 5 μm) used at a flow rate of 1 mL/min. Elution consisted of a linear gradient of the mobile phase: 0.3% formic acid in water (solvent A): acetonitrile (solvent B). Gradient elution using solvent B was as follows: 0–2 min at 10%, 2–15 min at 15%, 15–25 min at 35%, 25–35 min at 35%, and 35–40 min at 10%. UV detection at 280 and 350 nm was used. The same HPCL condition was applied for LW, but a different elution method was used: 1% formic acid and acetonitrile were used as mobile solvents A and B, respectively. The elution gradient of LW was as follows: 0–5 min at 5%, 5–20 min at 15%, 20–40 min at 35%, 40–50 min at 35%, 50–55min at 5%, and 55–60 min at 5%. SE and LW samples of the clarified filtrates were analyzed in triplicate. 

### 4.4. Cell Viability Assay

HepG2 cells (ATCC no. HB-8065) were obtained from the American Type Culture Collection (Rockville, MD, USA) and cultured in DMEM containing 100 mM sodium pyruvate, 10% fetal bovine serum, 10,000 U/mL penicillin and 10,000 ug/mL streptomycin. Cell culture was maintained at 37 °C in a humidified atmosphere of 5% CO2. On achieving 80–90% confluence, HepG2 cells were harvested using 0.25% trypsin-EDTA solution and mixed with complete DMEM medium.

The MTT assay is one of the frequently used assays for cell viability/cytotoxicity of plant extracts [55]. The ideal viability assay requires that the cells be safe, fast, reliable, efficient, and cost-effective, with no interference from any test compound [56]. Therefore, in this study, the cell viability of the LS extracts, Que, and GA on HepG2 cells was determined using MTT following a previous study [57]. HepG2cells were seeded in 96-well plates at a density of 2 × 10^4^ cells/well and cultured at 37 °C and 5% CO_2_ for 24 h. LS extracts, Que, and GA were dissolved in 50% DMSO and diluted with complete DMEM containing 0.1% DMSO in the culture medium. The compounds were diluted with medium at concentrations ranging from 1–1000 g/mL, and 10% dimethyl sulfoxide (DMSO) was added as a positive control for the 24-h exposure experiment. 

After removal of the medium, the cells were washed with PBS and incubated with 50 µL of 0.5% MTT solution for 2 h at 37 °C and 5% CO_2_. A total of 50 µL of DMSO was put into each well to dissolve the formazan crystals. The absorbance was detected using a microplate reader at 590 nm. The percentage cell viability was calculated according to the following equation:

$$\%\mathrm{Cell}\;\mathrm{viability}=\frac{A\;\left(\mathrm{sample}\right)-A\;\left(\mathrm{blank}\right)}{A\;\left(\mathrm{control}\right)-A\;\left(\mathrm{blank}\right)}\;\times \;100$$

where A (sample) is the absorbance value of the sample, A (blank) is the absorbance value of the blank, and A (negative control) is the absorbance value of the control (cells). The experiment was performed in triplicate. 

### 4.5. Animal Experiment

Male and female Wistar rats weighing 160–170 g were obtained from Nomura Siam International Co, Ltd., Bangkok, Thailand. All animals were housed in environmentally controlled conditions with a 12 h light/dark cycle and a temperature of 25 ± 1 °C. Food and water were freely available. All rats were allowed one week to acclimate to the housing conditions before starting the experiment. All experimental protocols were approved and operated by the Thammasat Animal Care and Use Committee, Thammasat University (No. 003/2561).

**Figure 8:**
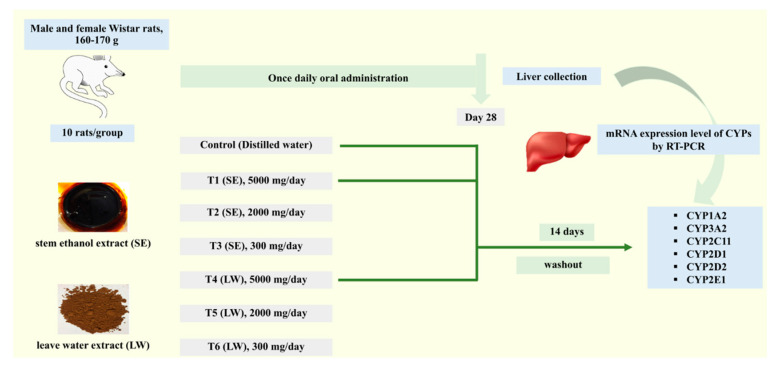
An overview of the animal experiment to investigate the effect of LS extracts on CYP450 mRNA expression in Wistar rat livers.

Rats were randomly assigned to seven experimental groups (n = 10). There were five rats of each gender per group. Rats were orally administered with SE or LW at doses of 300, 2000, or 5000 mg/kg.bw/day for twenty-eight days. Distilled water was given to the rats in a vehicle control group. At the end of the experimental period, the rats were sacrificed, and liver tissues were removed and immediately frozen and stored at −80 °C. In addition, the control group and SE-5000- and LW-5000-treated groups were extended as washout groups for fourteen days before euthanasia, and samples were collected for further analysis. The experimental procedure is shown in Figure 8. 

### 4.6. Total RNA Isolation and Quantitative RT-PCR (qRT-PCR)

Total RNA was isolated from tissues (approximately 50 mg) using Trizol reagent (Invitrogen, Camarillo, CA, USA) according to the manufacturer’s protocols. The total RNA concentration and purity were determined by measuring the absorbance at 260 and 280 nm using a NanoDrop 2000 (Thermo Scientific, Vilnius, Lithuania). RNA was transcribed into cDNA using the cDNA Synthesis Kit (Thermo Scientific, Vilnius, Lithuania). The obtained cDNA was stored at −20 °C for the qRT-PCR. In order to investigate the expression of the six CYP450 genes, i.e., CYP1A2, 3A2, 2C11, 2D1, 2D2, and 2E1, primers were designed and synthesized, as shown in Table 1. The qRT-PCR conditions were performed as follows: after pre-denaturation at 94 °C for 3 min, PCR was performed for 40 cycles, each of which consisted of 20 s at 94 °C, 20 s at 55 °C, and 30 s at 72 °C. Gene expressions were analyzed using the 2^–∆∆Ct^ method according to a previous study [58]. GAPDH was used as a housekeeping gene for the normalization of target gene expression. Every sample was analyzed in triplicate. 

### 4.7. Hierarchical Clustering Analysis (HCA) 

Six CYP450 genes expressed in the LS extract-treated rats were analyzed and clustered by HCA following the creation of a hierarchy group using Pearson’s correlation (average linkage clustering) in the software MultiExperiment Viewer (MeV) version 4.9.0 [59,60]. The gene expressions were presented in different color scales by a color bar in a heat map based on red (higher values), green (lower values), and black (intermediate values).

### 4.8. Statistical Analysis

Data were presented as the mean ± SEM and were analyzed using GraphPad InStat (GraphPad Software Inc., Version 8.0). One-way analysis of variance was used to detect significant differences between the control and treatment groups. Dunnett’s test was used for multiple comparisons. The differences were considered significant at *p* < 0.05.

## 5. Conclusions

**Figure 9:**
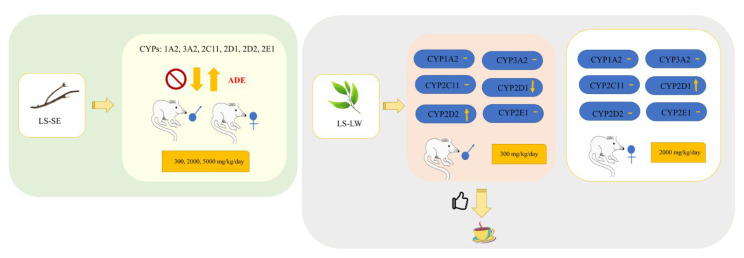
The effect of LS extracts on the hepatic transcription level of drug-metabolizing enzymes (CYPs450) in an animal study. ADE: Adverse Drug Effect.

To summarize, as shown in Figure 9, our study is the first to report the effects of LS extracts on the expression of six clinically relevant drug-metabolizing enzymes, i.e., CYP1A2, 3A2, 2C11, 2D1, 2D2, and 2E1, in animals. Our results revealed that in male rats given SE, daily doses of 2000 and 5000 mg induced CYP2C11, whereas the doses of 300 and 2000 mg induced CYP3A2. Only 2000 mg of SE could induce CYP2E1, and only 2000 mg of LW-contained GA could induce CYP2C11 and CYP1A2. CYP inhibition at the LW doses of 2000 for CYP3A2 and CYP2E1 and 5000 mg for CYP3A2 was also observed. By contrast, female rats given SE at all doses showed a dominant expression of CYP3A2 and CYP1A2. Interestingly, most doses of LW promoted the induction of CYP3A2, CYP1A2, and CYP2D1, which were mostly dominant at the highest dose of 5000 mg. Furthermore, 300 mg of LW stimulated the expression of CYP2E1 in female-treated rats. Our findings showed that the different active ingredients and doses of the LS extracts had a direct effect on CYP induction and inhibition in different genders, potentially causing HDIs. However, the mechanism of action of these extracts on nuclear factors should be further investigated.

## Figures and Tables

**Table 1:** Primers used in this study.

Gene	Primer Sequences (5’ ➔ 3’)	Size (bp)	Accession Number
CYP1A2	F: CGCCCAGAGCGGTTTCTTA	81	NM_012541.3
	R: TCCCAAGCCGAAGAGCATC		
CYP3A2	F: GGACTTAATTGACTGCTCTTGATG	222	NM_153312.2
	R: GGACGAGGACATGGTTACTATC		
CYP2C11	F: AGCTCTTGTTGATCTAGGAG	426	XM_008760364.2
	R: GGGAAGTAATCAATAATGGC		
CYP42D1	F: ACCCATGGCTTCTTTGCTTTTC	98	NM_153313.2
	R: CCTGTAGACTGGACTGGAA		
CYP2D2	F: CCAGGGCAACTTTGTGAAGC	193	NM_012730.2
CYP2E1	R: TGGAGTAACTGGCAATGCGT F: GCTGTCAAGGAGGTGCTAC R: GCCTCATTACCCTGTTTCC	182	NM_031543.1
GAPDH	F: TTCAACGGCACAGTCAAG	116	XM_017593963.1
	R: TACTCAGCACCAGCATCA		

## Data Availability

Data are contained within the article.

## Abbreviations

LS: *Lysiphyllum strychnifolium* (Craib) A. Schmitz; CYPs450, cytochrome P450s; SE, stem ethanol extract; LW, leaf water extract; Que, quercetin; GA, gallic acid; HDI, herb-drug interaction; bw, body weight; qRT-PCR, Quantitative RT-PCR; ADE, adverse drug effect; HPLC-DAD, High performance liquid chromatography photodiode array detection; SD, standard deviation; SEM, Standard Error of the Mean; HM, herbal medicine.
